# THE INTERSECTION OF INSOMNIA AND CENTRALIZED PAIN IN OLDER ADULTS

**DOI:** 10.1093/geroni/igad104.2804

**Published:** 2023-12-21

**Authors:** Michael Smith, Meagan Dean, Madison Irwin

**Affiliations:** University of Michigan, Ann Arbor, Michigan, United States; University of Michigan Health, Ann Arbor, Michigan, United States; University of Michigan, Ann Arbor, Michigan, United States

## Abstract

The intersection of insomnia and pain with age has been broadly studied but has not been evaluated specifically in centralized pain. The objective of this study was to estimate the effect of age on rates of centralized pain and insomnia in older adults. This was a single center, retrospective, observational cohort study consisting of older adults (≥55 years old) with a diagnosis of centralized pain, insomnia, or both seen at University of Michigan Health from January 1, 2016 – January 1, 2022. The primary outcome was the proportion of older adults with both insomnia and centralized pain, defined as least one centralized pain diagnosis coded in the subject’s electronic health record within one year of insomnia diagnosis. A total of 26,804 patients were included in the analysis. Mean age was 66.84 years (SD 8.79); 66% of patients (n=17,687) were female. Overall, 92.1% of patients (n=24,683) carried ≥1 centralized pain diagnosis, 5.6% of patients (n=1,505) carried a diagnosis of insomnia, and 2.3% (n=616) had both [95% CI 2.1,2.5]. Of older adults with insomnia, 29% (95% CI 27, 31) also carried a centralized pain diagnosis. Of older adults with centralized pain, 2.4% (95% CI 2.3, 2.6) also carried an insomnia diagnosis. Compared to older adults with insomnia alone, those with both insomnia and centralized pain were more likely to be female (p=< 0.001) and older (p=0.014). Insomnia and centralized pain are highly prevalent in older adults and co-occur frequently in those with insomnia. Older adults with insomnia should be screened for centralized pain.

